# HIV Treatment Proceeds as Prevention Research Confounds

**DOI:** 10.1371/journal.pmed.0040347

**Published:** 2007-12-01

**Authors:** The PLoS Medicine Editors

## Abstract

Introducing the World AIDS Day 2007 issue, The*PLoS Medicine* Editors call for continued efforts to develop definitive methods of HIV prevention.

World AIDS Day, the annual December 1 commemoration, first took place in 1988 under the auspices of the Joint United Nations Programme on HIV/AIDS [1]. At that time few had recognized the epidemic's impending global scope or envisaged how to provide AIDS treatment in developing countries. During the previous year, the United States Food and Drug Administration (FDA) had licensed zidovudine (AZT), the first drug shown to be effective for treating HIV. AZT, which quickly became available in North America and Europe, provided modest hope for the first time since the initial reports of AIDS in 1981. Taken as a single drug every four hours round the clock, it could prolong life by half a year or more [2].

Twenty years later, HIV treatment has the potential to become one of medicine's success stories. Combination anti-HIV therapies, referred to generically as highly active antiretroviral therapy (HAART), began to appear around 1996. Although costly and not without adverse effects, these “cocktails” have proved so effective that many who narrowly escaped death from AIDS in the mid-1990s are now facing the usual health concerns of advancing age. Although emergence of drug-resistant virus creates ongoing challenges, HIV treatment has continued to advance, as evidenced by the development of more convenient treatment regimens, and by FDA approval in 2007 of drugs from two new mechanistic classes: the first integrase inhibitor and the first chemokine receptor blocker.

On the global scale, 2007 has seen further progress in the impressive effort to address financial and logistical barriers to providing HIV treatment in low-resource settings. Although some 5 million people remain in need of HAART, and a recent systematic review found that many who begin HAART in developing countries do not continue zreatment [3], the fact that more than 2 million people in low- and middle-income countries are now receiving HAART marks significant progress.

In this context of global progress toward HIV treatment, the official theme of World AIDS Day 2007 appropriately calls on “Leadership” to “Keep the Promise” of universal access to HIV care and services.

Pounds of much-needed treatment, however, should not obscure the fact that precious ounces of prevention remain elusive: interrupting HIV transmission remains one of the world's greatest scientific challenges. Indeed, in contrast to the progress made in treatment, World AIDS Day 2007 marks the end of a particularly sobering year in HIV prevention science, particularly in the area of female-controlled methods, which have long been recognized as key to interrupting HIV transmission when social and economic disempowerment prevent women from insisting on condoms. January brought the announcement that two developing country trials of the vaginal microbicide cellulose sulfate had to be stopped because of an increased risk of HIV infection in women using the product [4,5]. The result was disappointingly reminiscent of the nonoxynol-9 vaginal microbicide trial that ended with a similar outcome in 2000 [6]. Another setback to female-controlled prevention came in July with the report that providing latex diaphragms and gel together with male condoms to women in southern Africa gave no additional protection against HIV compared with condoms alone [7]. September brought more bad news: the early cessation of a major international HIV vaccine trial when interim analysis found that the vaccine, Merck's trivalent adenovector product, appeared no better than placebo in preventing HIV infection [8]. Again the disappointment was familiar; in 2003 the first vaccine studies designed to assess protection against HIV in humans, using the VaxGen envelope products, were completed with no convincing evidence of efficacy.

In the area of behavioral prevention, a 2007 systematic review of available reports [9] found that abstinence-only programs, incorporated into many US and developing country HIV programs as a condition of US government funding, have been ineffective in reducing HIV risk in high-income countries. However, a study of abstinence-plus programs (which promote condom use as an alternative when abstinence fails) found the latter programs to be more promising [10]. In 2007, US-supported treatment programs directly or indirectly provided HAART to more than a million people in developing countries, and provided more general HIV care and prevention services to millions more. To assure success in HIV prevention, it is time for leaders of the US effort to act on the scientific evidence and end political requirements for abstinence-only funding.

In perhaps the year's most convincing results regarding HIV prevention, clinical trials in Uganda and Kenya, confirming an earlier trial from South Africa [11], showed that circumcision of adult men reduced their risk of acquiring HIV by about half over the subsequent two years [12,13]. However, even if this level of protection can be realized in the face of uncertain acceptance rates for circumcision and despite increased risk taking that may result from expectations of protection following circumcision, the risk reduction for a given male would still be no better than that of condom use for those who will use them. In turn, the effectiveness of circumcision—hardly a recent surgical technique—invites comparison to state-of-the-art research in immunology and virology, which have yet to deliver anything close to a reliable 50% reduction in risk of acquiring HIV through sexual exposure. Despite efforts of thousands of volunteers and expenditures of many tens of millions of dollars on clinical prevention trials ending in 2007, protection against sexual acquisition of HIV remains decidedly low-tech and frustratingly fixated on the phallus.

In terms of short-term benefit, then, it could be argued that basic research funding should instead be redirected toward condom education programs. In the long term, however, more definitive prevention methods are desperately needed to bring the AIDS crisis to an end, and we must not give up working toward a breakthrough in prevention comparable to the treatment advances of the past decade. New microbicides and vaccines are being developed and tested, trials of pre-exposure prophylaxis are under way, and more basic research in HIV epidemiology and pathogenesis continues to advance.

The essential need for global dissemination and discussion of research reports is nowhere better illustrated than in the response to the vast complexity of the AIDS pandemic. *PLoS Medicine* has published many papers on HIV/AIDS, is featuring several new papers [14–20] in this World AIDS Day issue, and will publish several more over the coming weeks. For announcements and discussion of these upcoming articles in *PLoS Medicine* as well as papers on HIV/AIDS in *PLoS ONE,* we invite our readers to check, and comment via, the *PLoS Medicine* blog (http://www.plos.org/cms/plosmedicine/).

## Abbreviations

AZT: zidovudine
FDA: Food and Drug Administration
HAART: highly active antiretroviral therapy
